# Quantifying Selection with Pool-Seq Time Series Data

**DOI:** 10.1093/molbev/msx225

**Published:** 2017-08-21

**Authors:** Thomas Taus, Andreas Futschik, Christian Schlötterer

**Affiliations:** 1 Institut für Populationsgenetik, Vetmeduni Vienna, Vienna, Austria; 2 Vienna Graduate School of Population Genetics, Vienna, Austria; 3 Department of Applied Statistics, Johannes Kepler Universität Linz, Linz, Austria

**Keywords:** evolve and resequence, time series, selection, software

## Abstract

Allele frequency time series data constitute a powerful resource for unraveling mechanisms of adaptation, because the temporal dimension captures important information about evolutionary forces. In particular, Evolve and Resequence (E&R), the whole-genome sequencing of replicated experimentally evolving populations, is becoming increasingly popular. Based on computer simulations several studies proposed experimental parameters to optimize the identification of the selection targets. No such recommendations are available for the underlying parameters selection strength and dominance. Here, we introduce a highly accurate method to estimate selection parameters from replicated time series data, which is fast enough to be applied on a genome scale. Using this new method, we evaluate how experimental parameters can be optimized to obtain the most reliable estimates for selection parameters. We show that the effective population size (*N*_e_) and the number of replicates have the largest impact. Because the number of time points and sequencing coverage had only a minor effect, we suggest that time series analysis is feasible without major increase in sequencing costs. We anticipate that time series analysis will become routine in E&R studies.

## Introduction

The underlying molecular mechanisms of adaption in natural populations to novel environments have been of long-standing interest in evolutionary genetics. Nevertheless, it is also becoming increasingly clear that information about adaptive alleles provides an enormous potential for a broad range of disciplines including vaccine and drug development (e.g., Andries etal. 2005; Sequist etal. 2011), animal and plant breeding (e.g., Hufford etal. 2012; Daetwyler etal. 2014), pest management, biomedicine and many more. A widely used approach for the identification of selection relies on pattern of sequence variation, which are not compatible with the null hypothesis of neutral drift, preferentially accounting for past demographic processes. One of the most simple selection signatures is generated by a beneficial allele sweeping through the population; linked neutral alleles are hitchhiking with the target of selection (Smith and Haigh 1974; Barton 2000) generating patterns of reduced variability, increased linkage disequilibrium and skewed site frequency spectra around the targeted genomic region. These signals are generated by sequence variants flanking the selected site and provide inference power for popular tests for selection (e.g., Tajima 1983; Fay and Wu 2000; Kim and Stephan 2002; Sabeti etal. 2002; Kim and Nielsen 2004; Jensen etal. 2005; Nielsen etal. 2005; Voight etal. 2006; Foll and Gaggiotti 2008; Pavlidis etal. 2013; Ferrer-Admetlla etal. 2014). While these tests utilize polymorphism data from a single time point, the analysis of samples collected during multiple time points may be powerful enough to infer selection for a single site taking advantage of the allele frequency changes across the different time intervals. Although the advantage of time series for population genetic analyses has been appreciated for a long time (Fisher and Ford 1947; Wright 1948), due to the lack of adequate data this approach had not been receiving much attention. With the arrival of second-generation sequencing the situation has changed and with decreasing sequencing costs, the collection of time series data has become feasible and is being recognized as a powerful approach to study adaptive processes.

One recent example for the successful use of time series data is the sequencing of ancient human remains to characterize trajectories of selected alleles and to identify candidate loci in European human populations (Mathieson etal. 2015). Another study of seasonal population samples collected over 3 years demonstrated that natural *Drosophila melanogaster* populations rapidly respond to selection driven by seasonal changes in the environment (Bergland etal. 2014). Nevertheless, one particular challenge for the inference of adaptive processes from natural populations arises from demographic processes, which may result in biased estimates if not accounted for (e.g., Thornton etal. 2007; Crisci etal. 2012; Haasl and Payseur 2016).

An alternative source of high resolution time series data comes from experimental evolution studies combined with sequencing pools of individuals (Pool-Seq) (Futschik and Schlötterer 2010; Schlötterer etal. 2014), which provide replicated samples at multiple time points. Because the environmental conditions are well controlled and evolutionary trajectories can be monitored in replicates, such evolve and resequence (E&R) studies (Turner etal. 2011) are particularly informative to link selection signatures and adaptive changes to a given environment. Over the past years adaptation to novel environments was studied within an E&R framework in a wide range of organisms, including bacteria (e.g., Barrick etal. 2009; Woods etal. 2011; Tenaillon etal. 2012, 2016), viruses (e.g., Foll etal. 2014), yeast (e.g., Kao and Sherlock 2008; Lang etal. 2013; Burke etal. 2014; McDonald etal. 2016), *Drosophila* (e.g., Burke etal. 2010; Turner etal. 2011; Zhou etal. 2011; Orozco-terWengel etal. 2012; Turner and Miller 2012; Tobler etal. 2014; Griffin etal. 2017), and mice (e.g., Chan etal. 2012).

Despite the conceptual appeal and an increasing number of suitable data sets, the inference of selection parameters from time series data remains a significant challenge. Unlike in cases where random drift can be ignored (Illingworth etal. 2012), calculating the exact probability of allele frequency trajectories for finite population sizes comes at a huge computational cost (Jewett etal. 2016). Hence, transition probabilities are approximated (Terhorst etal. 2015; Topa etal. 2015; Khatri 2016) and parameters are estimated in a maximum likelihood (ML) (Bollback etal. 2008; Malaspinas etal. 2012; Mathieson and McVean 2013; Steinrücken etal. 2014; Iranmehr etal. 2017) or a Bayesian framework (Ferrer-Admetlla etal. 2016; Schraiber etal. 2016). Because these methods are still computationally rather demanding, estimates were also obtained by forward simulations in combination with approximate Bayesian computation (Foll etal. 2015). While all these methods account for random allele frequency fluctuations owing to finite population sizes, some are capable of estimating both *N_e_* and *s* (Bollback etal. 2008; Malaspinas etal. 2012; Steinrücken etal. 2014; Foll etal. 2015; Ferrer-Admetlla etal. 2016; Iranmehr etal. 2017), whereas others rely on independent *N_e_* estimates (Mathieson and McVean 2013). Recently, Jewett etal. (2016) suggested that purely deterministic models, despite ignoring the effects of random drift, could provide accurate estimates and reduce the computation time by several orders of magnitude. This implies that the characterization of selection dynamics for millions of loci genome-wide is possible in a reasonable time frame. However, it remains unclear if accuracy is sufficiently high, when such deterministic models are applied to E&R studies in sexual organisms, such as *Drosophila*, where *N_e_* is in the hundreds (e.g., Tobler etal. 2014). Additionally, available methods are mostly limited to the analysis of single trajectories, while state-of-the-art E&R studies generate replicated allele frequency trajectories. Thus, the inference potential provided by modern experimental designs is often not fully exploited.

Several computer simulation studies provided guidelines about the experimental design of E&R studies to optimize the detection of selected loci (Baldwin-Brown etal. 2014; Kofler and Schlötterer 2014; Kessner and Novembre 2015). In contrast, not much is known about the influence of the experimental design on the inference of selection parameters. We introduce a fast and highly accurate approach to estimate the selection coefficient *s* and dominance *h* and evaluate different designs for E&R. We show that the number of replicates and the effective population size are the primary factors determining the accuracy of estimates. Because a large number of time points and a high sequencing depth are not needed in terms of accuracy, time series analyses are affordable and will most likely be the standard for future E&R analyses.

## Results and Discussion

We introduce a new approach to estimate *s* for haploid and diploid populations that combines information of replicated evolutionary trajectories. In its basic form, it employs linear least squares regression (LLS) to fit allele frequency data to a purely deterministic selection model.

### Robustness under a Wide Range of Scenarios

**Fig. 1.**
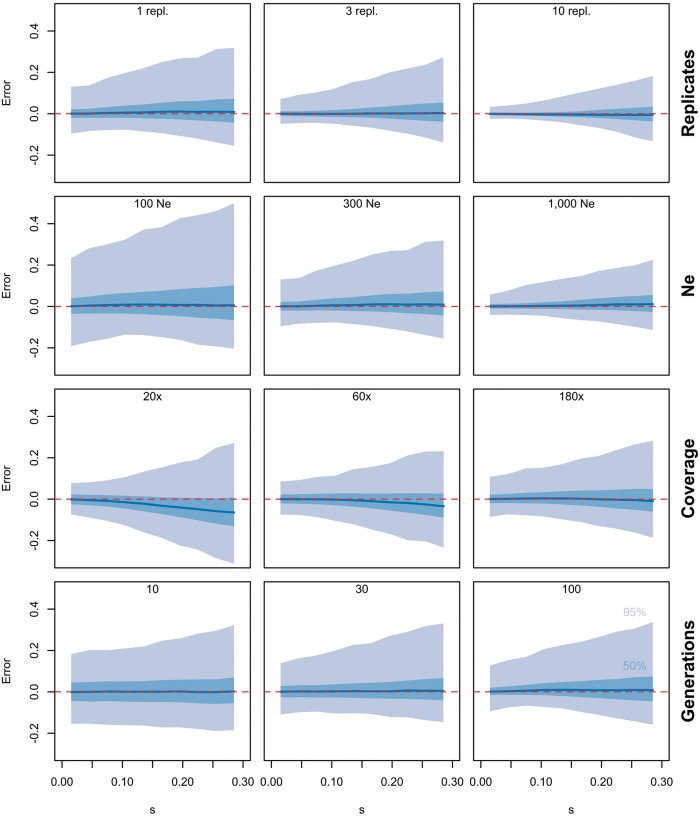
Robustness of LLS under a wide range of scenarios. Allele frequency trajectories were simulated for 100,000 unlinked loci. Unless noted otherwise the default parameters were 60 geneations, *N_e_ *= 300, *p_0_* ∈ [0, 1] and *s* ∈ [0, 0.3] and 1 replicate. We tested the influence of the number of replicates, *N_e_*, sequencing coverage or the total number of generations (values on top of each panel) on the robustness of LLS. A bias correction was applied to all estimates (see Materials and Methods). The absolute error between estimated and true *s* is shown: median (dark blue), 25–75% quantile (light blue) and 2.5–97.5% quantile (gray) of values.

We tested the robustness of the LLS method by applying it to a set of 1.2 million simulated trajectories varying *s* and *p_0_*. These simulations covered a wide range of experimental setups differing in the number of replicates, *N_e_*, sequencing coverage and the total number of generations. Selection coefficients were estimated with LLS and contrasted with the actual parameter values (fig. 1). *N_e_* and the number of replicates had the largest effect on the precision of selection estimates, whereas sequencing coverage and number of generations only played a minor role. Although the precision varied across experimental parameters, LLS-based estimates were unbiased under all scenarios examined. For low sequencing coverage (20×) only, our selection estimators showed some bias with values that were systematically too small. This bias appears to be negligible compared with the low precision that results from the sampling noise. Interestingly, despite this drop in precision for low sequence coverage, our method was still powerful to identify selected loci (87%, α = 0.01).

We related the performance of our approach to WFABC (Foll etal. 2015) and CLEAR (Iranmehr etal. 2017). The former was previously favorably compared to other methods estimating *s* from time series data—in particular for accuracy under strong selection and computational efficiency (Foll etal. 2015). CLEAR is a method to detect selection in E&R data (Iranmehr etal. 2017), which also estimates *s*. We simulated allele frequency trajectories for 100,000 loci and 60 generations with *p_0_* ∈ [0, 1] and *s* ∈ [0, 0.3] assuming *N_e_* = 300. Allele frequencies were sampled every ten generations mimicking an average Pool-Seq sequencing depth of 80×. The strength of selection was estimated for single trajectories only assuming *h* = 0.5, with LLS, WFABC, and CLEAR. Accuracy was measured by the relative root–mean–square error (rRMSE, see Materials and Methods for details), since this measure captures both bias and variance of an estimator.

**Fig. 2.**
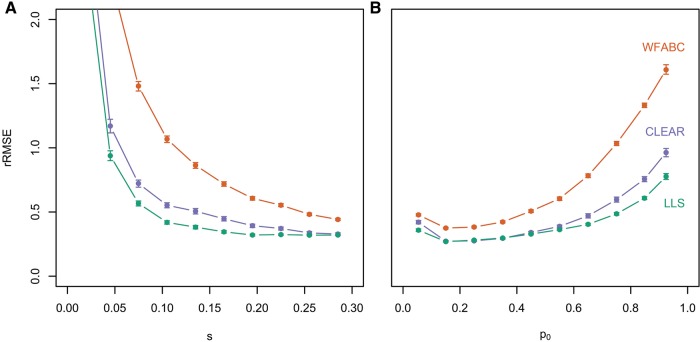
Comparison of LLS, WFABC, and CLEAR. Allele frequency tajectories were simulated for 100,000 unlinked loci over 60 generations assuming *N_e_ *= 300 with *p_0_* ∈ [0, 1] and *s* ∈ [0, 0.3]. Selection coefficients were estimated with LLS (green), as well as WFABC (orange), and CLEAR (purple). The relative deviation between estimated and true selection coefficient is shown as a function of *s* (*A*) and *p_0_*(*B*).

All methods provided the most reliable estimates for large selection coefficients (see fig. 2A) and intermediate (between 10% and 30%) starting allele frequencies (see fig. 2B). With decreasing selective advantage, estimates became less accurate, but LLS-based estimates provided lower rRMSE values than those of CLEAR and WFABC. The accuracy dropped (and rRMSE increased) also with increasing starting allele frequency for all methods, but the effect was less pronounced for LLS and CLEAR. Under weak selection, as well as high starting allele frequency of the beneficial allele, it is challenging to disentangle directional selection from random drift. Our results suggest that the good performance of LLS becomes particularly evident for those cases where it is challenging to distinguish selection from drift.

Applying any selection inference procedure on a genomic scale requires a computationally effective method. It has recently been suggested that runtimes of ML-based estimators can be reduced by several orders of magnitude by ignoring demographic histories, while maintaining high accuracy if *N_e_* and *s* are large enough (Jewett etal. 2016). WFABC is considerably faster than several ML-based approaches (Foll etal. 2015), but we found that LLS required on average 0.0015 s per locus, while CLEAR and WFABC needed about 0.1 and 1.4 s, respectively. For LLS this translates into a processing time of 25 min for one million loci. Notably, the processing time of CLEAR increased dramatically with *N_e_*, while it remained fairly constant with LLS (see supplementary table S1, Supplementary Material online). Most importantly, the higher computational efficiency of LLS does not come at the cost of reduced accuracy; as for challenging scenarios (small *s* or large *p_0_*) it is more accurate than WFABC, while providing a level of accuracy similar to CLEAR.

**Fig. 3.**
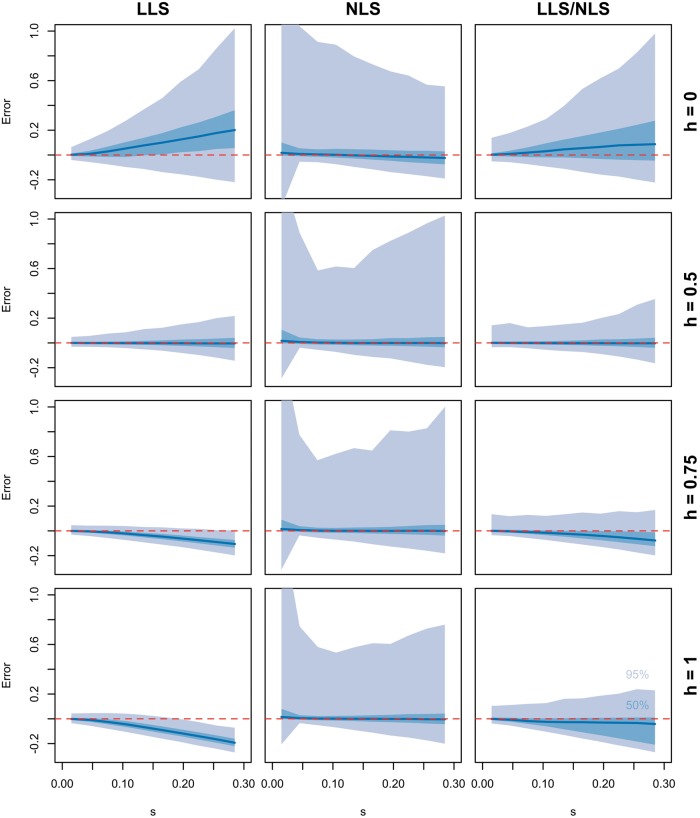
Impact of the dominance parameter on the accuracy of selection estimates. Allele frequency trajectories were simulated in six replicates for 100,000 unlinked loci over 60 geneations assuming *N_e_ *= 300 with *p_0_* ∈ [0, 1] and *s* ∈ [0, 0.3]. The dominance parameter (*h*) was assumed to be 0, 0.5, 0.75, or 1. Selection coefficients were estimated with both LLS and NLS. A bias correction was applied to all estimates (see Materials and Methods). Additionally, an automatic switching approach was employed (LLS/NLS) that uses NLS-based estimates instead of those of LLS, if there is sufficient statistical evidence for the need of a more complex selection model. Illustrated is the median (dark blue), 25–75% quantile (light blue) and 2.5–97.5% quantile (gray) of absolute errors between true and estimated selection coefficient as a function of *s*.

So far, we assumed codominance (*h *= 0.5), but since dominance has a pronounced effect on the frequency trajectory of selected alleles, we tested the robustness of LLS with different degrees of dominance. We simulated replicated allele frequency trajectories with *h* ∈ {0, 0.5, 0.75, 1} and estimated selection coefficients with LLS. While *s* is upward biased for recessive alleles, we notice a downward bias in the case of dominance (see fig. 3).

These results emphasize that ignoring dominance could result in highly biased results with the LLS method. Using the nonlinear least squares regression (NLS) approach to coestimate *s* and *h* from time series data, we obtain unbiased selection coefficient estimates for all values of *h* (see fig. 3). While the width of the 50% band of errors is comparable between LLS and NLS, we obtain more outliers with NLS, which is reflected in a rather broad 95% band of errors. Analyzing the 1% most extreme outliers suggests that that the numerical procedure to optimize the sum of squared errors is robust with respect to the starting value and therefore multiple local optima appear to be only rarely responsible for the reduced precision (see supplementary fig. S1, Supplementary Material online). Rather, it seems that outliers occur frequently, when *p_0_* of the beneficial allele is high and the respective trajectories are not informative enough to coestimate *s* and *h* (see supplementary figs. S2 and S3, Supplementary Material online).

To combine the precision of LLS with the robustness of NLS, we developed a procedure, which relies on NLS estimates only when the data suggest a deviation from codominance. We inferred the deviation from codominance by adding a quadratic term to the linear model. If the *P* value of the quadratic term is sufficiently small, NLS is used instead of LLS. A too stringent threshold implies that LLS will be preferred, which will provide potentially biased estimates, if the assumption of *h* = 0.5 is violated. On the other hand, too liberal *P* value thresholds favor NLS, which is in general less precise but always unbiased. Using a *P* value threshold of 0.1, our results suggested that our automated switching method constitutes a good compromise between precision and bias (see fig. 3). The specificity of the switching procedure appeared to be best for *p_0_* < 1/3 (see supplementary fig. S4, Supplementary Material online). In the case of higher starting allele frequencies, trajectories may not be informative enough to detect deviations from codominance and estimates may be biased.

### Benefit of Replicated Allele Frequency Trajectories

Genetic drift and sampling noise during allele frequency measurements (e.g., Pool-Seq) are responsible for deviations between true and estimated parameter values. The former can be reduced either by increasing *N_e_* or by inferring parameters using the information from multiple evolutionary trajectories simultaneously, as available in experimental evolution with replicate populations. The latter can be decreased by increasing pool size and sequencing depth. This raises the question whether multiple replicates or increased sequencing coverage are more effective to improve our estimates. To address this, we simulated time series data under two scenarios with the same sequencing effort: 1) single trajectories with high sequencing coverage (480×) and 2) six replicates with 80× each. An effective population size of 300 was chosen, because it reflects a realistic value for E&R studies in sexual organisms, such as *Drosophila*. Selection coefficients were estimated with LLS.

**Fig. 4.**
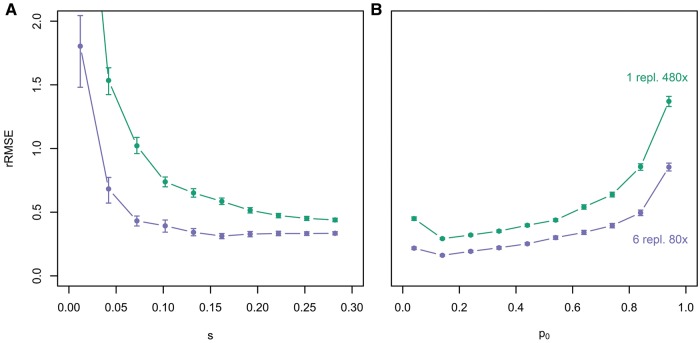
The advantage of replication over high sequencing coverage. Allele frequency tajectories were simulated for 100,000 unlinked loci over 60 generations assuming *N_e_* = 300 with *p_0_* ∈ [0, 1] and *s* ∈ [0, 0.3]. Selection coefficients were estimated with LLS using either a single replicate per locus (green), or by combining the information of multiple replicates (purple). The overall sequencing effort was assumed to be constant. The relative deviation between estimated and true selection coefficient is shown as a function of *s* (*A*) and *p_0_*(*B*).

With the same sequencing effort, we found for the entire parameter space that more replicates provided more accurate results than a single replicate with higher sequencing coverage (see fig. 4). This result makes intuitive sense, because by combining allele frequency estimates across independently evolved replicates the effect of random drift is reduced, while increasing the read depth reduces solely the loss in accuracy due to the sequencing noise. This emphasizes the importance of sequencing multiple replicates, when studying populations with small *N_e_*. It is important to note that the reduction in rRMSE by increasing the number of replicates will be less pronounced for large *N_e_*, for example, in E&R studies with microbes, because single trajectories will be close to deterministic ones.

### Performance under a Typical E&R Scenario

We further characterized the accuracy of *s* estimates, by simulating allele frequency trajectories for unlinked loci under experimental parameters that resemble those of recent E&R studies with *Drosophila* (e.g., Turner etal. 2011; Tobler etal. 2014; Griffin etal. 2017). For a total of ten million loci, six independent trajectories were simulated over 60 generations each with population allele frequency estimates at every 10th generation. Sampling noise was added to mimic Pool-Seq with an average sequencing coverage of 80×. The entire parameter space was split into 2,500 equally sized bins based on both *p_0_* and *s*, and accuracy was assessed based on the rRMSE.

**Fig. 5.**
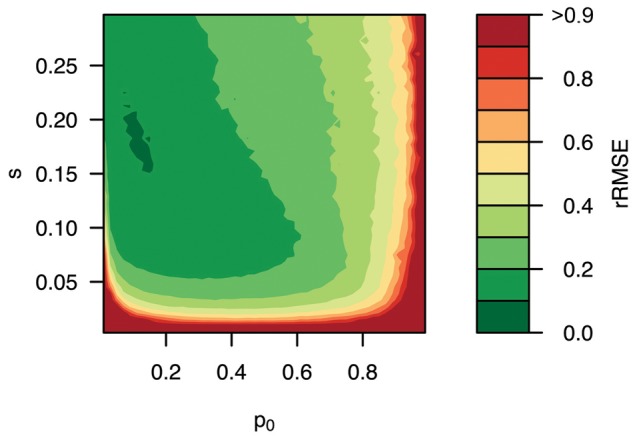
Accuracy of selection coefficient estimates under a state-of-the-art E&R experiment. Allele frequency trajectories were simulated in six replicates for ten million unlinked loci over 60 geneations assuming *N_e_ *= 300 with *p_0_* ∈ [0, 1] and *s* ∈ [0, 0.3]. Selection coefficients were estimated with LLS. Estimates were divided into 2,500 bins based on *p_0_* and *s*, before computing the rRMSE for each bin. Illustrated are the color-coded rRMSE values for each region of the parameter space, with green and red corresponding to high and low accuracy, respectively.

For 60% of the parameter space, estimates of *s* were highly accurate with rRMSE below 0.3 (see fig. 5). Notably, this included selection coefficients as low as 0.05, if the beneficial allele started at a frequency between 0.1 and 0.7. For low starting frequencies (*p_0_* < 0.1) and weak selection (*s* < 0.05) the estimates were less accurate. In general, rRMSE exceeds 0.9 if either *p_0_* > 95% or *s* < 0.01. High starting frequencies result in short and uninformative trajectories, which translates in the higher uncertainty. If the fitness benefit is too small, it is challenging to distinguish random drift from selection and the relative error of estimates increases.

Analyzing the same data set with the automatic switching method provided comparable results for variants under strong selection (*s* > 0.1) with a starting allele frequency below 60% (see supplementary fig. S5, Supplementary Material online). For rare alleles under weak selection, or variants at high frequency (*p_0_* > 0.75) estimates were imprecise and the rRMSE exceeded 0.9.

**Fig. 6.**
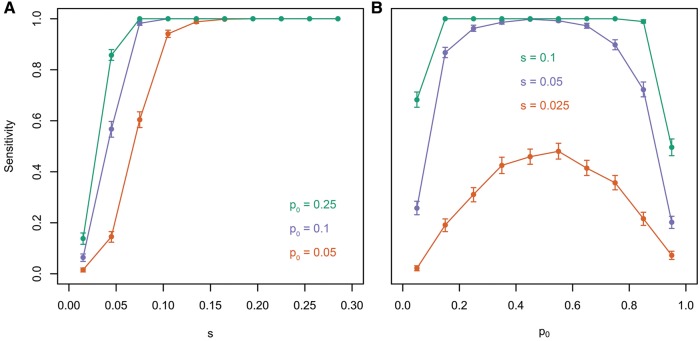
Sensitivity to identify selected loci. Allele frequency trajectories were simulated in six replicates for 10,000 unlinked loci over 60 geneations assuming *N_e_* = 300 varying both *p_0_* and *s*. Selection coefficients were estimated and the null hypothesis of random drift was tested with LLS. Illustrated is the sensitivity in identifying selected loci (α = 0.01) as a function of *s* and *p_0_*. (*A*) Beneficial variants started at 5% (orange), 10% (purple) or 25% (green) with *s* ∈ [0, 0.3]. (*B*) The selection advantage was equal to 0.025 (orange), 0.05 (purple), or 0.1 (green) with *p_0_* ∈ [0, 1].

Genetic drift results in random allele frequency changes, thus time series data could distinguish between random (genetic drift) and directional (selection) allele frequency changes. Nevertheless, by chance genetic drift can also generate time series data that resemble those of selected alleles. We used computer simulations to distinguish selection from neutral expectations under drift. Specifically, we determined the probability of a given selection coefficient under the null hypothesis of random genetic drift by parametric bootstrapping (see Materials and Methods). Applying this neutrality test to the aforementioned simulations we determined the fraction of significant loci (α = 0.01)—that is, the estimated power of our test—for different values of *s* and *p_0_* (see fig. 6).

LLS-based selection tests turned out to be very powerful. More than 80% of the selected loci with *s* > 0.03, *s* > 0.06, or *s* > 0.09 were detected for starting allele frequencies of *p_0_* = 0.25, *p_0_* = 0.1, or *p_0_* = 0.05, respectively. Fitness advantages as low as 2.5% can be detected in >40% of the cases if *p_0_* is between 0.3 and 0.7. Variants with 5% selective advantage can be detected in >80% of the cases if they start between 0.1 and 0.8. Thus, even under challenging conditions, selected loci can be detected with a state-of-the-art E&R design at reasonable sensitivity using our method. However, linkage between loci and competition between beneficial loci in close proximity (Hill and Robertson 1966) will complicate the identification of selection targets.

These results also emphasize that the detection limit of weakly selected alleles can be improved by avoiding small starting allele frequencies. Although this may seem trivial it is tricky to achieve. On one hand, starting the experiment from a diverse set of haplotypes is beneficial because levels of linkage disequilibrium (LD) are low (Baldwin-Brown etal. 2014; Kofler and Schlötterer 2014; Kessner and Novembre 2015). On the other hand, under such an experimental setup, most alleles will be at low frequency, thus increasing the number of founder chromosomes will also increase the probability of low frequency alleles under selection.

**Fig. 7.**
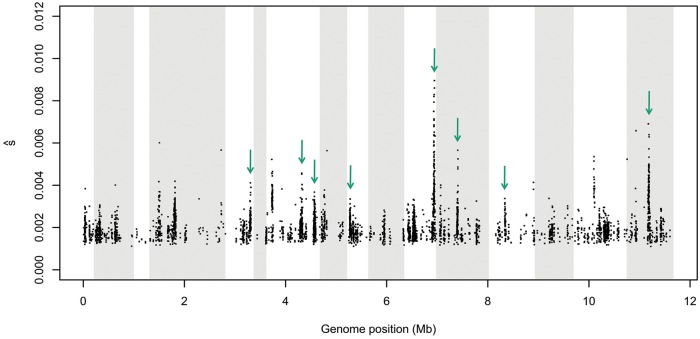
Genome-wide quantification of selection coefficients. The time series data of a recent E&R study in yeast (Burke etal. 2014) were analyzed with LLS. Selection coefficients are plotted for significant SNPs (FDR 0.01) with the *X*-axis specifying their genomic positions in Mb. Different shadings of gray illustrate individual chromosomes. Green arrows mark genomic regions that were identified as targets of selection in the original analysis.

Recently, a highly outcrossed diploid *Saccharomyces cerevisiae* population originating from four founder genotypes was exposed to competitive growth in liquid media for 540 generations (Burke etal. 2014). Small starting allele frequencies were avoided, because the founding population was derived from four haplotypes only. Strain specific alleles occurred on average at a frequency around 25%. Still, levels of LD were kept low owing to the intense outcrossing scheme between the four founder haplotypes. Such starting frequencies greatly facilitate the identification of targets of selection, even for weakly selected sites (see also fig. 6). Using LLS with an FDR of 0.01, we identified all selected genomic regions of the original analysis and inferred selection coefficients as low as 0.002 (see fig. 7). Compared with the previous study, we found more candidate loci, which we attribute to differences in statistical power between the methods, particularly when selection is acting on alleles with extreme *p_0_* (see supplementary fig. S6, Supplementary Material online). Notably, a rather complex life cycle was imposed in this E&R study and it is unclear if assumptions of a constant selective pressure underlying our analytic model are met. With a considerable number of generations of asexual growth, clonal interference could further complicate the analysis. Thus, we caution that deviations from the model assumptions—if present—may have influenced the inferred selection coefficients and the exact number and location of true targets of selection require further investigation.

### Impact of Experimental Parameters

**Fig. 8.**
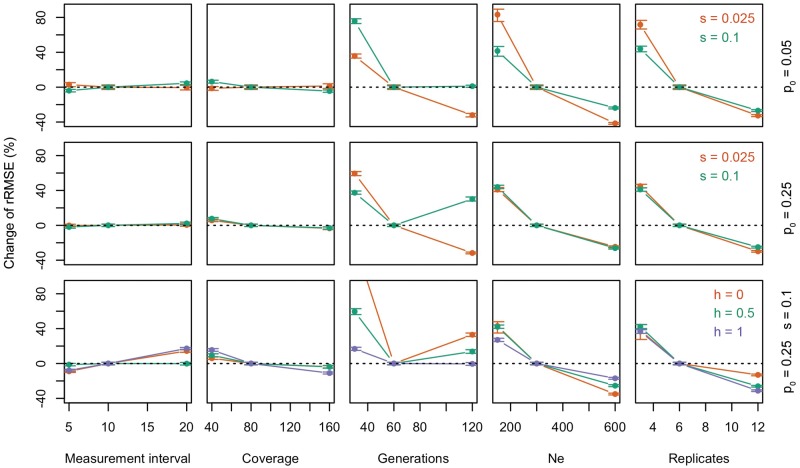
Impact of experimental parameters on the accuracy of *s* estimates. Allele frequency trajectories were simulated in six replicates for 10,000 unlinked loci over 60 geneations assuming *N_e_* = 300 for rare (*p_0_* = 0.05) and common (*p_0_* = 0.25) alleles under weak (*s* = 0.025) or strong (*s* = 0.1) selection. Additionally, we also varied the dominance parameter between 0, 0.5, and 1. We tested the influence of the measurement interval, sequencing coverage, total number of generations, *N_e_*, and the number of replicates on the accuracy of *s* estimates. Illustrated is the change of rRMSE in percent, when modifying one of the experimental parameters.

Previous studies evaluating the experimental design of E&R studies (Baldwin-Brown etal. 2014; Kofler and Schlötterer 2014; Kessner and Novembre 2015) focused on the power to identify selection targets, but did not evaluate the influence of the experimental design on the inference of selection parameters. Thus, we evaluated the impact of several experimental parameters on the accuracy of estimated selection coefficients. Allele frequency trajectories were simulated for weak (*s* = 0.025) and strong (*s* = 0.1) selection on rare (*p_0_* = 0.05) or common (*p_0_* = 0.25) variants under the standard E&R scenario (300 *N_e_*, 6 replicates, and 60 generations, measuring allele frequencies every ten generations at 80× coverage). The selection coefficient *s* was estimated with LLS. Furthermore, we studied the influence of dominance (for *p_0_* = 0.25 and *s* = 0.1) using the switching method. The performance of the estimation procedure was measured again using the relative root mean squared error rRMSE, since it captures both precision and bias (see fig. 8).

The measurement interval determines the temporal resolution of allele frequency trajectories. Keeping the total number of generations constant, shorter intervals result in higher resolution and more measurements. Altering the time interval between 5, 10, and 20 generations, which correspond to 13, 7, and 4 allele frequency measurements, respectively, did not change the accuracy of *s* estimates. Already four time points (F0, F20, F40, and F60) resulted in accurate selection coefficient estimates. Unlike codominant targets of selection, the analysis of dominant and recessive alleles benefits from shorter measurement intervals. Similarly, sequencing coverage played a minor role regarding the accuracy of selection estimates and already a coverage of 40× produced reasonable estimates. This is in contrast to analyses performed for two time points, where sequencing coverage was identified as a key factor for reliable identification of selection targets (Kofler and Schlötterer 2014). We conclude that the availability of multiple time points compensates for the increased sampling variance at lower coverage.

The duration of the experiment had a pronounced effect on the accuracy. While for weakly selected alleles rRMSE decreased continuously with the duration of the experiment, for strongly selected alleles the optimal rRMSE was seen for an intermediate number of generations. For strongly selected alleles (*s* = 0.1) with a starting frequency of 0.25, the rRMSE increased even when trajectories covered 120 generations. Deterministic trajectories show that under strong selection (*s* = 0.1) on a rare variant (*p_0_* = 0.05), allele frequencies start to plateau at generation 90, while they keep increasing under weak selection (*s* = 0.025, see supplementary fig. S7, Supplementary Material online). This is even more extreme for beneficial variants that are common (*p_0_* = 0.25, see supplementary fig. S7, Supplementary Material online). Hence, monitoring alleles under strong selection for a large number of generations may not provide additional information and the accuracy of selection estimates does not improve (see supplementary fig. S8, Supplementary Material online). It is important to note that for both LLS and the automatic switching method, reducing the number of generations from 60 to 30 severely deteriorated the accuracy of *s*-estimates for all parameter tested.

Independent of *p_0_*, *s*, and *h, N_e_* and the number of replicates had the most pronounced influence on accuracy. At low starting frequencies, weakly selected variants benefited more than strongly selected ones from more replicates or larger *N_e_*. Under codominance both *N_e_* and the number of replicates had similar effects, but for recessive alleles (*h* = 0) the influence of *N_e_* was more pronounced and for dominance (*h* = 1) increasing the number of replicates had a larger effect.

While *N_e_* and number of replicates were the parameters with the largest potential to improve E&R studies, we also caution that the costs associated with either increased population size or more replicates may be a limiting factor—in particular for studies with a substantial phenotyping component.

### Analyzing Time Series Data with the poolSeq R-Package

We implemented the methods described here in the user-friendly R-package poolSeq (available at https://github.com/ThomasTaus/poolSeq). The major functions of poolSeq are the simulation Pool-Seq data for unlinked loci, *N_e_* estimation (Jónás etal. 2016) and the inference of selection parameters. Forward in time simulations are performed for unlinked loci of haploid or diploid individuals under a Wright–Fisher model. Both the selection coefficient and dominance parameter can be specified. Sampling noise can be added to allele frequencies to mimic the Pool-Seq process. Alternatively to simulating data, allele counts can be loaded into R from the commonly used “sync” file format (Kofler etal. 2011). The package also provides fast implementations of the chi-squared and Cochran–Mantel–Haenszel test.

## Materials and Methods

### Estimating *s* and *h* from Time Series Data

We consider unlinked biallelic loci with alleles *A* and *a* that evolve at a constant population size *N_e_* for *t* nonoverlapping generations. Relative fitness is given by *w_A_*=1 + *s* and *w_a_*=1 for the two alleles in haploids. In the diploid case, the relative fitness of the three possible genotypes *AA*, *Aa*, and *aa* is defined as *w_AA_* = 1 + *s*, *w_Aa_* = 1 + *hs* and *w_aa_* = 1. Allele frequencies are denoted *p* and 1-*p* for *A* and *a*, respectively. In populations of infinite size the allele frequency after one generation of selection (*p’*) can be computed for haploid individuals as:

(1) $$p^{\prime }=\frac{w_{A}p}{w_{A}p+w_{a}\left(1-p\right)}$$

and for diploid individuals as:

(2) $$p^{\prime }=\frac{w_{AA}p^{2}+w_{Aa}p\left(1-p\right)}{w_{AA}p^{2}+w_{Aa}2p\left(1-p\right)+w_{aa}{\left(1-p\right)}^{2}}.$$

Using a continuous time approximation with overlapping generations and assuming weak selection, the trajectory of the selected allele for *h* = 0.5 (Crow and Kimura 1970) in infinite haploid populations is given by:

(3) $$p\left(t\right)=\frac{1}{1+\left(\frac{1-p_{0}}{p_{0}}\right)e^{-st}}.$$

The corresponding formula for diploids is:

(4) $$p\left(t\right)=\frac{1}{1+\left(\frac{1-p_{0}}{p_{0}}\right)e^{\frac{-st}{2}}}.$$

From this a linear relationship between logit-transformed allele frequencies and the number of generations is obtained, both in the haploid and in the diploid case:

(5) $$\ln\left(\frac{p\left(t\right)}{1-p\left(t\right)}\right)=\ln\left(\frac{p_{0}}{1-p_{0}}\right)+st,$$

(6) $$\ln\left(\frac{p\left(t\right)}{1-p\left(t\right)}\right)=\ln\left(\frac{p_{0}}{1-p_{0}}\right)+\frac{s}{2}t.$$

Using equations (5) and (6), we can infer both *s* and *p_0_* by fitting a linear model with least squares regression (LLS) to logit-transformed allele frequency trajectories. The slope of the linear model provides the selection coefficient, while the starting allele frequency is given by the intercept. However, with increasing strength of selection, the discrepancy between the discrete and continuous model grows (see supplementary fig. S9, Supplementary Material online), ultimately biasing the selection coefficient estimates for populations evolving in discrete generations. We therefore propose a bias correction, in cases where the estimated selection coefficient is large. This is done by adding the difference between the selection coefficient used to compute a deterministic allele frequency trajectory and the value obtained by reestimating the parameter with LLS (see equation [5] or [6]).

It turns out that the logit transform stabilizes the drift variance (see supplementary fig. S10, Supplementary Material online). If sequencing coverage varies across the time points, the fit to the regression model could be further improved in principle by taking nonhomogeneous variances into account. This would imply to fit a weighted linear model, with the inverse coverage as weights. As shown by our simulations however (see fig. 1), the sequencing coverage does not affect the accuracy of estimation much, unless it is very small.

Relaxing the assumption of codominance (*h *= 0.5) requires a more general function describing the allele frequency depending on *p_0_*, *s*, *h*, and *t*. Although a general solution to the underlying differential equation (Crow and Kimura 1970) is available, numerical methods are required to estimate both *s* and *h* for a given allele frequency trajectory. Therefore, we coded a function to perform discrete forward in time computations based on equations (1) and (2) to obtain deterministic allele frequency trajectories. This function was used within the nonlinear least squares routine *nls* in R, to obtain parameter estimates for both *s* and *h*. While this approach worked for a broad range of parameter combinations, the algorithm did not converge in a few cases (see supplementary fig. S11, Supplementary Material online).

To rule out that an estimated selection coefficient could be obtained by random genetic drift, we simulate neutral (*s *= 0) allele frequency trajectories and estimate *s* with either LLS or NLS. The *P* value for the null hypothesis of neutrality is the fraction of simulations resulting in estimates of *s*, which are at least as large as the selection coefficient inferred from the empirical data.

Assuming that *s* and *h* do not change, replicated trajectories improve the inference when the information of all replicates is combined. Assuming that all replicates start from the same allele frequencies, we averaged the allele frequencies at each time point across replicates. In the case of small *N_e_*, selected alleles with a low starting frequency have a high probability of being lost which would bias the inferred selection parameters. We therefore excluded observed allele frequencies in a replicate from the time an allele gets lost onwards. In cases where the starting allele frequency is low compared to *N_e_*, this conditioning on fixation needs to be accounted for. We therefore propose for a further bias correction in such cases, that is based on neutral simulations for each locus individually, leading to corrected empirical consensus trajectories. The corrected consensus trajectories can then be used in such cases for LLS- and NLS-based inference of *s* (and *h*).

### Precision and Bias of Estimates

To assess the precision and bias of estimated selection coefficients *s*, we used the relative root–mean–squared error (rRMSE), a measure of the relative deviation between estimated and true selection coefficient. It was computed for bins of *n* loci:

(7) $$rRMSE=\frac{\sqrt{\frac{\sum_{i=1}^{n}{\left({\hat{s}}_{i}-s_{i}\right)}^{2}}{n}}}{\overset{-}{s}},$$

where ${\hat{s}}_{i}$ is the estimated and $s_{i}$ is the true selection coefficient of locus *i*, and $\overset{-}{s}$ is the mean selection coefficient of all *n* loci. In the comparison between LLS, CLEAR, and WFABC, estimates were binned according to either *s* or *p_0_* and then the rRMSE was computed for each bin. Similarly, when assessing the accuracy of LLS under a state-of-the-art E&R design, ten million estimates were divided into 2,500 bins depending on *s* and *p_0_*. This way, the dependence of the rRMSE on *s* and *p_0_* has been illustrated. Confidence intervals of rRMSE were estimated by bootstrapping loci in each bin.

### Benchmark between LLS, CLEAR, and WFABC

We compared LLS-based estimates of *s* to those obtained with CLEAR (downloaded from https://github.com/airanmehr/clear on May 5, 2017) and WFABC (version 1.1) using the same allele frequency trajectories. To match the default parameter setting of WFABC, only trajectories with a minor allele frequency larger than 0.01 at any of the time points were used. We provided the true effective population size to WFABC (fixed_N 600). A flat prior between −0.2 (min_s) and 1.0 (max_s) was used. For CLEAR, *N_e_* was set to 300 (–N 300) and the maximum selection coefficient was set to 1, scanning in steps of 0.01 (maxS = 1 and stepS = 0.01). We further specified that all 300 individuals were sampled using Pool-Seq (*n* = 300).

The maximal deviation of ABC-based estimates from the true value depends on the range of the prior distribution, while LLS estimates could theoretically range from negative and positive infinity. To compare the methods, we projected LLS and CLEAR estimates to the range of the priors of WFABC.

Allele frequency trajectories were simulated under the Wright–Fisher model for diploid individuals assuming linkage equilibrium between loci. Starting allele frequencies were drawn from a uniform distribution between 0 and 1 and selection coefficients were between 0 and 0.3. To mimic sampling noise of Pool-Seq, first sequence coverage for each locus was drawn from a Poisson distribution and then binomial sampling was performed with sample size matching coverage.

### Analysis of E&R Study in Yeast

Allele frequency trajectories of all SNPs were downloaded from http://wfitch.bio.uci.edu (last accessed April 10, 2017). Without further filtering, selection coefficients were estimated and *P* values under the null hypothesis of random genetic drift were computed with LLS for all 75,410 loci. We assumed a population of haploid individuals, since the lifecycle imposed in this E&R study involved haploids for most of the time. We used the Benjamini–Hochberg correction (Benjamini and Hochberg 1995) to correct the *P* values for multiple hypothesis testing. We only considered outliers, which were supported by at least one additional outlier SNP within 1 kb.

## Supplementary Material

Supplementary data are available at *Molecular Biology and Evolution* online.

## Supplementary Material

Supplementary Data
