# Supplementary material for: Quantifying Selection with Pool-Seq Time Series Data
Source: Mol Biol Evol. 2017 Aug 21;34(11):3023–34. doi: 10.1093/molbev/msx225 (PMC5850601; doi:10.1093/molbev/msx225)
Supplement: Supplementary Data [file msx225_supp.pdf]

Supplementary Results

Table S1: Runtimes in seconds per locus for LLS, CLEAR and WFABC. Allele frequency trajectories were simulated for 10,000 loci assuming 100/300/1,000 N_e, p_0 = 0.05 and drawing *s* from a uniform distribution between 0 and 0.3. Then *s* was estimated with LLS, CLEAR and WFABC and the average runtime in seconds per locus was computed. WFABC was applied to 100 trajectories only to reduce the overall processing time.

	**Ne = 100**	**Ne = 300**	**Ne = 1,000**
LLS	0.0014	0.0015	0.0015
CLEAR	0.04	0.10	0.77
WFABC	1.3	1.4	1.5

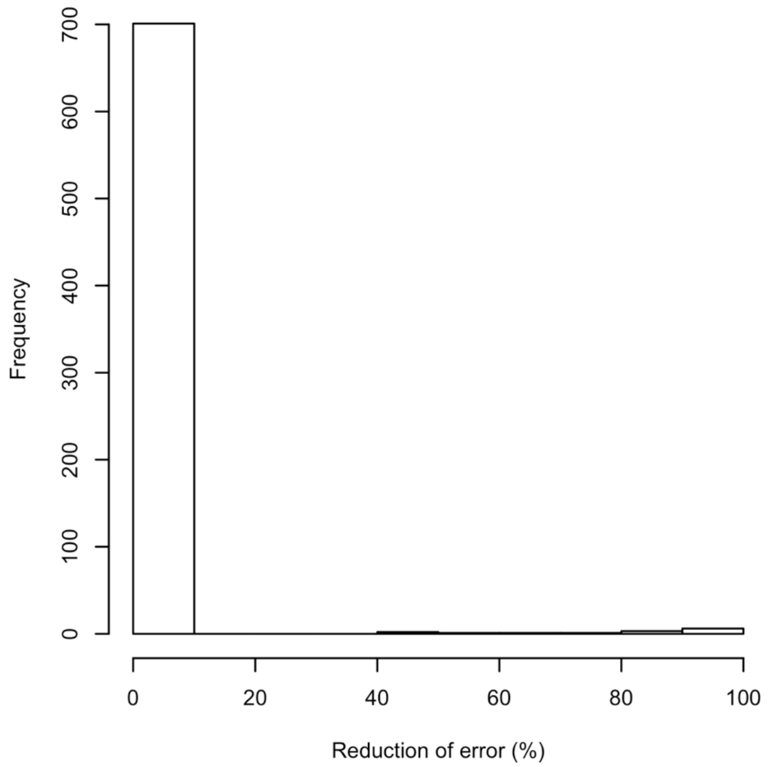

Figure S1: Robustness of NLS to local optima. Selection coefficients and dominance parameters were re-estimated with NLS for simulated trajectories which gave rise to extreme (top 1%) errors in Fig. 3. To estimate parameters, NLS by default optimizes the sum of squares starting with $s = 0.1$ and $h = 0.5$. Here, different starting values were used for s and h, and the relative improvement based on the most accurate estimate is shown. For the vast majority of the 715 considered outlier loci, no or only little improvement was possible.

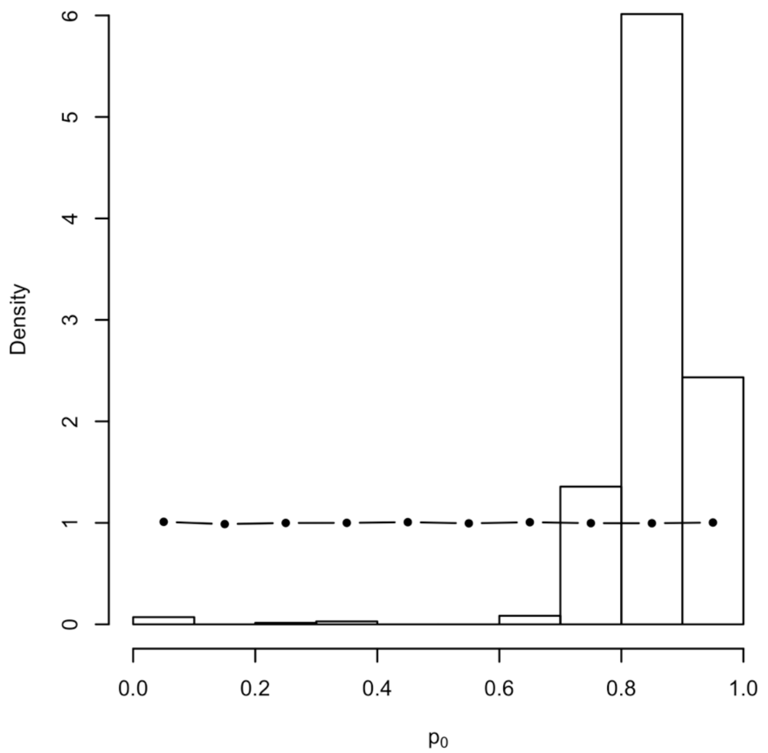

Figure S2: Extreme outliers with NLS were mostly encountered with large starting allele frequencies. The distribution of p_0 is shown for trajectories that resulted in extreme (top 1%) outliers with NLS in the simulations leading to Fig 3. The black reference line displays the uniform distribution of p_0 across all 100,000 simulated loci.

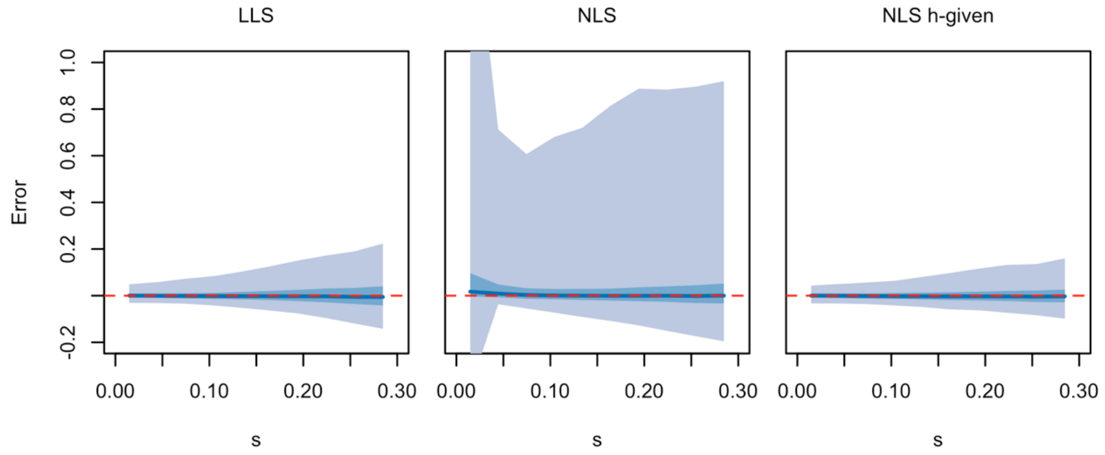

Figure S3: Precision of NLS improves dramatically when *h* is given. Allele frequency trajectories were simulated in six replicates for 100,000 unlinked loci over 60 geneations assuming N_e = 300 with $p_0 \in [0,1]$, $s \in [0,0.3]$ and $h = 0.5$. Selection coefficients were estimated with LLS and NLS, with the latter co-estimating h. Additionally, we applied NLS assuming that $h = 0.5$ is known (NLS h-given). The median (dark blue), 25% to 75% quantile (light blue) and 2.5% to 97.5% quantile (grey) of absolute errors between true and estimated selection coefficient are shown as a function of *s*. The figure suggests that it is considerably more difficult to estimate *s* and *h* simultaneously.

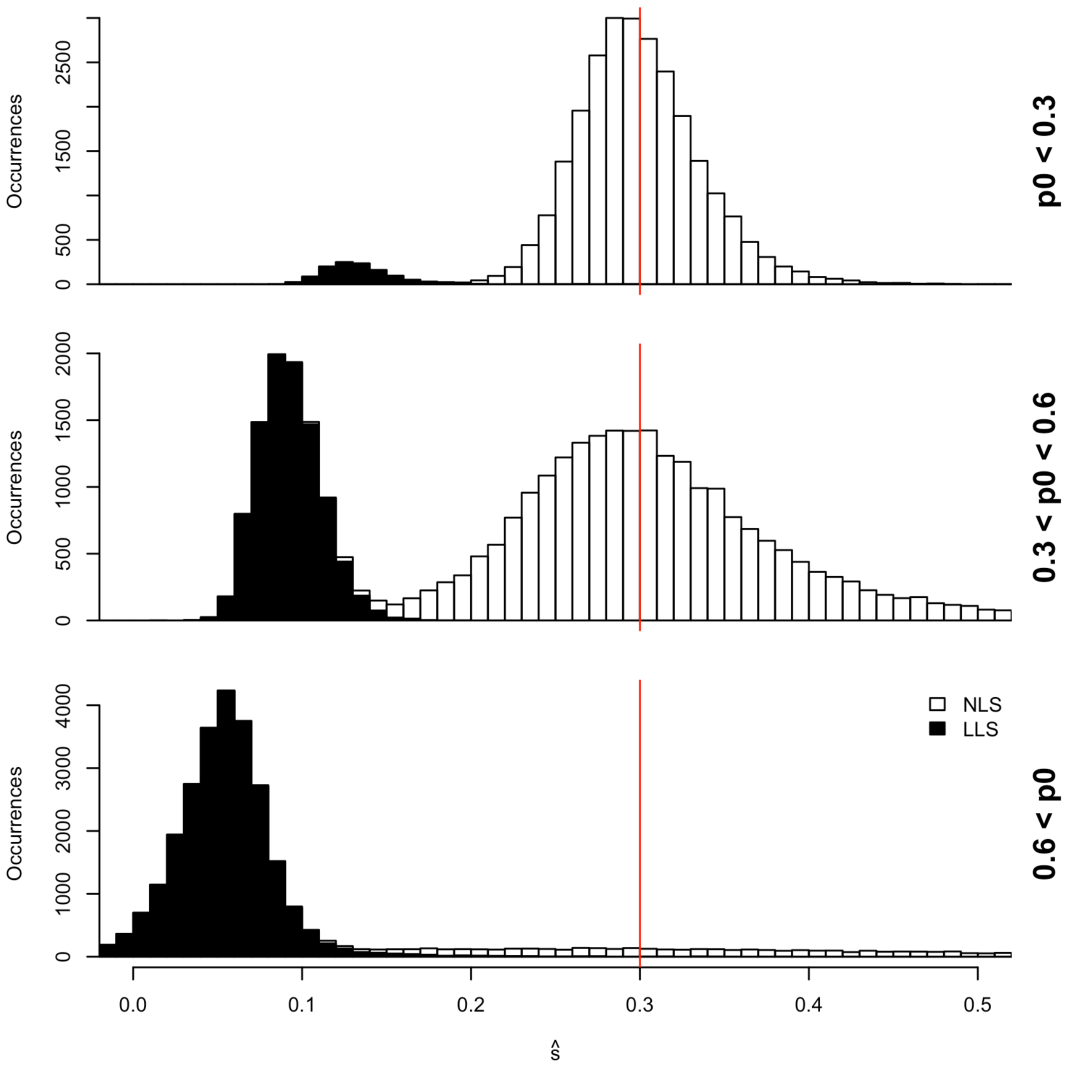

Figure S4: Specificity of automated switching between LLS and NLS best for p_0 < 0.3. Replicated allele frequency trajectories were simulated with s = 0.3 and h = 1. Selection coefficients were estimated automatically switching between LLS and NLS. The distribution of estimates obtained is shown for different ranges of p_0. The red line indicates the true parameter value.

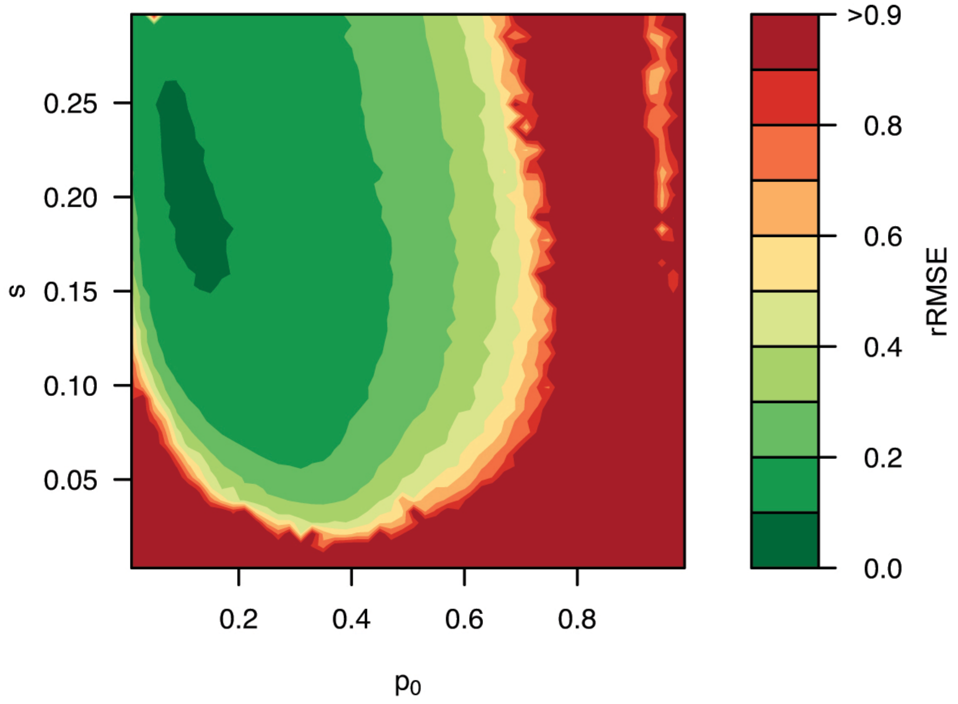

Figure S5: Accuracy of *s*-estimates with the automatic switching method. Allele frequency trajectories were simulated under an E&R scenario and selection coefficients were estimated automatically switching between LLS and NLS. The accuracy of the method was quantified based on the CV.

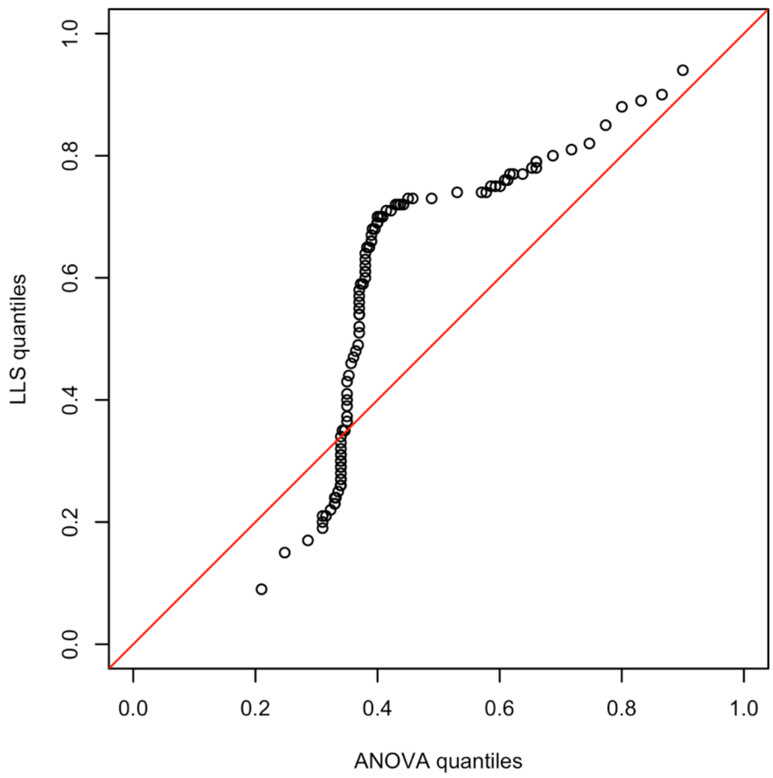

Figure S6: LLS provides more power to detect selected alleles with low and high *p_0*. We show a QQ-plot to compare the distributions of starting allele frequencies between significant loci identified with LLS and those reported in supplementary table S2 of Burke et al. 2014 (ANOVA).

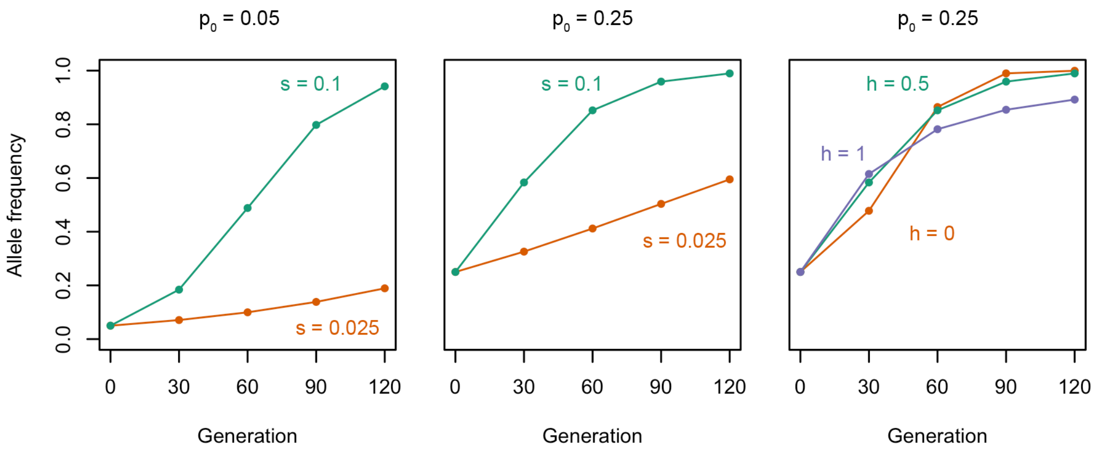

Figure S7: Deterministic allele frequency trajectories. Considering an infinitely large population, allele frequency trajectories were computed for various combinations of *s*, p_0 and *h*.

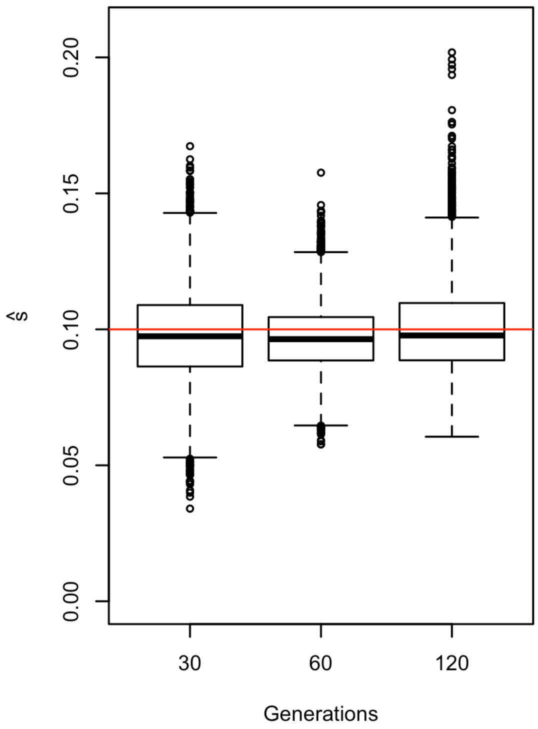

Figure S8: Under strong selection accuracy stagnates with increasing number of generations. Allele frequency trajectories were simulated for s = 0.1 and p_0 = 0.25 for 30, 60 and 120 generations. Then s was estimated with LLS.

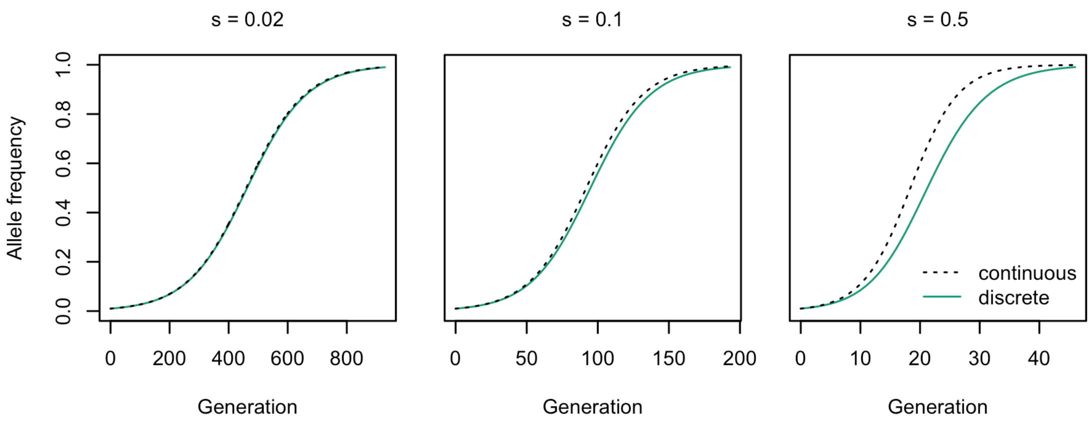

Figure S9: Deviation between discrete and continuous models for various strengths of selection. Assuming an infinitely large population, selection on an allele starting from 1% was simulated using both a discrete and continuous model (overlapping generations). Allele frequency trajectories for selection coefficients of 0.02, 0.1 and 0.5 were simulated.

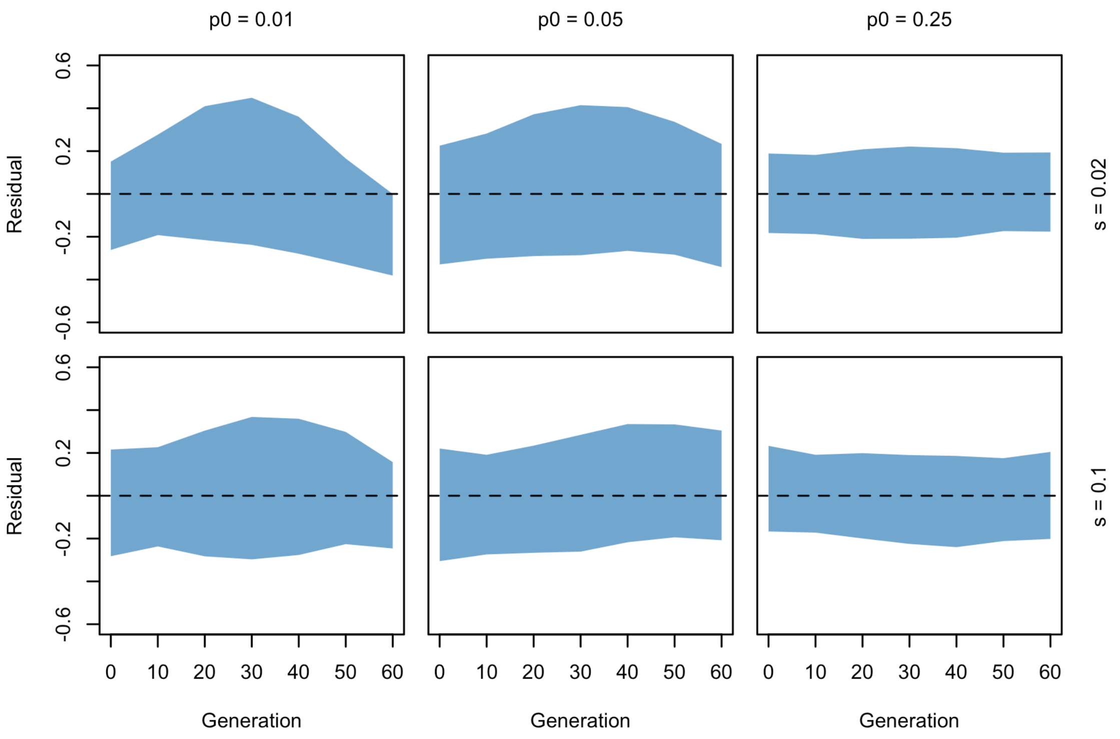

Figure S10: Residual error of LLS. Allele frequency trajectories were simulated for different values of p_0 and s, assuming that Ne equals 300. The residual error (50% band) is shown after fitting a linear least squares model to logit-transformed allele frequencies.

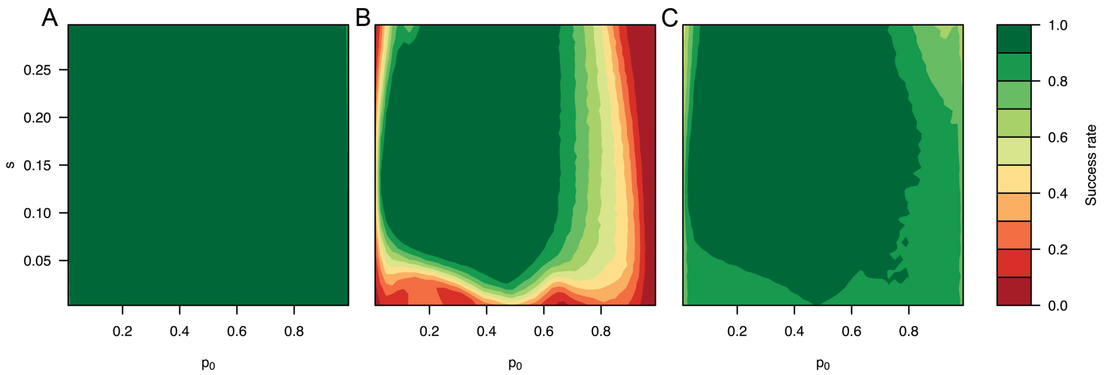

Figure S11: Success rate of LLS, NLS and the autmoatic switching method under a state-of-the-art E&R experiment. Allele frequency trajectories were simulated in six replicates for 10 million unlinked loci over 60 geneations assuming N_e = 300 with $p_0 \in [0,1]$ and $s \in [0,0.3]$. Selection coefficients were estimated with LLS (A), NLS (B) or the automatic switching method (C). Estimates were divided into 2,500 bins based on p_0 and s. For each bin the fraction of loci, for which selection estimates could be obtained, (success rate) was computed. Illustrated are the color-coded success rates for each region of the parameter space, with green and red corresponding to high and low rate of success, respectively.
